# Physiological Responses to Repeated Maximum Intensity Efforts in Surface and Underwater Fin Swimming

**DOI:** 10.5114/jhk/199380

**Published:** 2025-07-21

**Authors:** Ioannis D. Kostoulas, Gregory Kalaitzoglidis, George Tsalis, Konstantina Karatrantou, Argyris G. Toubekis, Vassilis Gerodimos

**Affiliations:** 1 Department of Physical & Cultural Education, Hellenic Army Academy, Athens, Greece.; 2 Department of Physical Education & Sport Science, University of Thessaly, Trikala, Greece.; 3 Department of Physical Education & Sports Science, Aristotle University of Thessaloniki, Serres, Greece.; 4 Department of Physical Education & Sport Science, National & Kapodistrian University of Athens, Athens, Greece.

**Keywords:** respiratory function, dynamic apnea, underwater fin swimming, surface fin swimming

## Abstract

The purpose of the study was to compare physiological and performance responses during surface and underwater fin swimming. Thirteen male, elite fin swimmers performed four repetitions of 50-m sprints using a monofin (4 x 50 m) either on surface (S) or underwater with apnea (U). Performance time and the number of lower body kicks were recorded during 4 x 50-m sprints. Lactate was evaluated before the start, after the second and the fourth sprint and five minutes into recovery. The heart rate was measured continuously and respiratory function was recorded before and after each condition. Performance time was shorter and kicking frequency was higher in U compared to S (17.73 ± 1.18 vs. 19.94 ± 1.41 s, 135 ± 18 vs. 121 ± 15 kicks∙min^−1^, p < 0.05). Lactate concentration was no different between conditions. Forced expiratory volume in 1 s and peak expiratory flow were no different before and after the sprints and between conditions (p > 0.05). Forced vital capacity and maximal voluntary ventilation were increased after sprints under both conditions (p < 0.05). The heart rate was decreased in U compared to S during both sprints (167–177 vs. 183–185 b∙min^−1^) and the recovery period (141–151 vs. 166–174 b∙min^−1^). Underwater and surface repeated sprint swimming present maximal dynamic and physiological responses that should be considered during fin swimming training.

## Introduction

Sprint fin swimming is a demanding competitive event that can be conducted either on the surface of the water using a snorkel or underwater and involves the use of monofins on the legs (*CMAS*, 2023). The 50-m event requires skilled swimmers to handle not only the monofin up and downward movements, but also the dynamic apnea required in the underwater event. Fin swimming applied in repeated efforts during a training session places high physiological demands on swimmers, including metabolic heart rate responses and respiratory function (Guimard et al., 2014).

The swimming speed in the underwater fin swimming with the monofin was reported to be 5.6% higher than on the surface (Nicolas and Bideau, 2009). A higher maximal speed at a given distance will reduce the time required to cover the distance increasing anaerobic contribution (Ogita, 2006). Thus, short duration training sets of repeated fin swimming efforts may induce different physiological and adaptive responses when using surface or the underwater technique and this has not been examined using fin swimming. In terms of kinematics, the self-selected kicking frequency has been reported as a determinant factor in sustaining monofin swimming speed at submaximal intensity (Vercruyssen et al., 2012). Moreover, maximum intensity underwater fin swimming presents lower kicking frequency, a higher vertical amplitude and lower active drag, compared to surface fin swimming (Nicolas and Bideau, 2009). These changes during repeated sprint fin swimming have not been reported previously.

Moreover, to achieve high level performance under apnea conditions, it is essential to develop physical adaptations and improve underwater swimming technique. The duration of apnea is determined by the intensity of exercise, with higher intensity resulting in shorter apnea time (Lin et al., 1974). Research in the field of apnea training has shown that a protocol of repeated apnea efforts can progressively improve the duration of apnea and may be used as priming to improve performance (Bourdas and Geladas 2021, 2024).

During both static and dynamic apneas, the reduced peripheral oxygen transport caused by intense peripheral vasoconstriction and the magnitude of the diving reflex may be related to the increased anaerobic metabolism and subsequent lactate levels (Andersson et al., 2004). Accordingly, reduced frequency breathing in high intensity swimming can lead to an increase in lactate concentration (Woorons et al., 2010).

The heart rate (HR) is an essential factor in evaluating and monitoring swimmers' performance during maximal repetitive effort training (Botonis et al., 2022) and may be used to determine the intensity of effort and predict any potential health issues that athletes may experience during training (Halson, 2014). Additionally, there is a need to conduct an objective assessment of high-intensity efforts using the rating of perceived exertion (RPE) scale (Borg, 1982; Möller et al., 2022).

Besides kinematic, metabolic and perceptual responses that may differ between surface and underwater fin swimming, it is the respiratory system that may be substantially taxed with apnea. Athletes who train under apnea conditions experience a slowdown in oxygen transport in the blood, which causes the body to manage oxygen stores more efficiently, prolonging the apnea time (Ferretti et al., 1991), while chemical stimuli such as hypoxia or hypercapnia may facilitate adaptations (Lemaître et al., 2002; Matúš et al., 2024).

Monofin swimmers frequently perform short-distance swimming intervals at maximum effort, either on the water's surface or underwater, followed by moderate recovery periods (Stavrou et al., 2015). This demanding training regime requires a high level of skill and it is interesting to assess swimmers' responses on surface and underwater. Therefore, the aim of this study was to examine the effects of repeated maximal intensity efforts during fin swimming training on performance, metabolic, respiratory and perceived responses at surface and underwater.

## Methods

Thirteen male monofin swimmers (n = 13, age: 17.3 ± 2.7 years, body height: 176 ± 7 cm, body mass: 70.6 ± 16.4 kg, body fat content: 12.5 ± 4.6%) participated in the study. All of them were national champions, six of them were members of the national team and among the six best athletes in Europe and the world, winning several medals in European and World championships the year that the study was completed. All participants and their guardians were informed in detail about the experimental procedures and signed an informed consent statement. Participants were selected for inclusion if they took part in daily training and were able to successfully complete 4 x 50-m fin swimming efforts with monofin in total apnea and with no respiratory problems. The study was approved by the Internal Ethics Committee of the Department of Physical Education and Sports Science of the University of Thessaly, Greece (approval code: 1830; approval date: 13 October 2021) and conformed to the Helsinki declaration for human subjects.

### Experimental Procedures

Anthropometric characteristics were determined for all participants. On following days, they performed four 50-m swimming maximum-intensity efforts with monofin (4 x 50-m) under two different conditions: i) on the surface (S), using a snorkel, and ii) underwater (U) (total apnea). Recovery time between each effort was 2 min allowing completion of the demanding apneic set and providing adequate but not complete restoration of energy stores (Bogdanis et al., 1998). The two measurements were performed 48 hours apart. The experiment took place over two weeks before the summer national championships. The order of the tests was counterbalanced and all procedures were performed at the same time of the day. Participants were encouraged to apply maximum effort from the first, up to the last 50-m sprint. All sprints started with a push-off from within the water in a 50-m outdoor swimming pool.

Before the start and at the fourth minute after the end of each condition, respiratory variables, forced expiratory volume in 1 s (FEV1), forced vital capacity (FVC), peak expiratory flow (PEF) and maximal voluntary ventilation (MVV) were measured. The fourth minute was used to allow completion of blood sampling along with collection of expired air measurement and appropriate positioning of the participants out of the water. Peak values for oxygen uptake (V̇O_2peak_), carbon dioxide production (V̇CO_2_) and pulmonary ventilation (V̇E) were determined by the collection of the expired air before and immediately after each condition at the end of the fourth sprint with swimmers in a standing position inside the swimming pool. Participants were asked to breathe into a mouthpiece immediately after a nose clip was fitted on. The time from the end of the test until breathing into the mouthpiece did not exceed 2–3 s. Time to complete each sprint and the number of lower body kicks were recorded. A capillary blood sample was collected at the start, after the second and the fourth sprint as well as during the fifth minute of recovery, to determine lactate concentration using a portable analyzer (Lactate Scout, SensLab GmbH, Leipzig, Germany). The fifth minute sample was used to secure the peak lactate concentration after the sprints (Kounalakis et al., 2014). The HR was recorded continuously using a chest belt (Polar H10; Polar Electro, Finland) that was placed under the CMAS approved full body competitive swimsuit, used in all tests, and remained attached to the chest without any movement. The RPE was reported after each sprint under both conditions using the Borg's 10-grade scale (Borg, 1982).

A controlled 20-min warm-up was applied before each session and a 10-min recovery was allowed after the warm-up before the commencement of the first sprint under each condition. The water temperature was kept at 24–25°C and the air temperature was 25°C on both days of measurements. Before the first trial, athletes made themselves familiar with all the equipment. Participants were asked to follow similar activities and apply the same diet with controlled carbohydrate content two days before each experimental session. They were also advised to avoid heavy exercise, alcohol or caffeine consumption for 24 hours preceding each session. All athletes used homologated and certified monofin by CMAS (Gold Fins, Gold Fins - Zolotov, Ukraine).

### Analytical Methods and Equipment

Standing height was recorded (Seca 206; Seca, Hamburg, Germany) and body mass was measured using an electronic precision scale (Seca 813; Seca), with athletes wearing a swimsuit. Body fat content was evaluated via the bioelectrical impedance method (Akern, BIA 101; Akern, Pontassieve, Italy). After a rest period of 20 min with the participant in the supine position, self-adhesive electrodes were fitted on the right hand and foot as per manufacturer’s instructions, and body fat content was evaluated. Participants abstained from the intake of coffee for at least 2 hours before the body fat percentage estimation process.

A portable gas analyzer (MicroQuark, Cosmed, Rome, Italy) was used to measure forced expiratory volume in 1 s (FEV1), forced vital capacity (FVC), peak expiratory flow (PEF) and maximal voluntary ventilation (MVV). The measurements were performed pre and post each condition according to the instructions of the ATS/ERS (Miller, 2005). The measurement of oxygen uptake, carbon dioxide production and pulmonary ventilation was conducted with a portable analyzer (PNOE, ENDO Medical, Palo Alto, CA). For consistency, gas calibration was completed with a 16% O_2_ and 4% CO_2_ standard gas tank. No flow meter calibrations were needed. The peak values of the above variables were determined through the retro-extrapolation method in the first linear segment of recovery (20 s). The retro-extrapolation methods overestimated the criterion values by 4–14% and the single yielded an underestimation of 3.4% (Kostoulas et al., 2021; Rodríguez et al., 2017). In the present study, the values corresponding to the first 3 s that were extrapolated from the linear fit of the relationship between V̇O_2_ and recovery time were used (Kostoulas et al., 2021; Rodríguez et al., 2017).

Performance time to complete each 50-m sprint was recorded by three experienced certified timekeepers and the three obtained values were averaged. The inter-individual difference in the time score was always under 3%. The completion times of the tests were measured with digital hand stopwatches (SL 929-R, Oregon Scientific, Tualatin, OR, USA). The number of lower body kicks in each sprint was counted and the kick frequency was calculated.

### Statistical Analyses

Statistical analysis was applied using Statistica 10.0 (StatSoft, Inc., Tulsa, OK, USA). A two-way ANOVA (2 conditions x 4 sprints) with repeated measures and a Bonferonni post-hoc comparison was performed. The data sphericity was checked with the Mauchly test. Under no circumstances was there a violation of sphericity. Additionally, the partial effect size η^2^, which takes values between 0 and 1, was calculated as a measure of the effect size and interpreted as small (below 0.02), moderate (0.02 to 0.13), and large effect size (> 0.26). The results are presented as mean value ± standard deviation, unless otherwise defined. The significance level was set at *α* ≤ 0.05.

## Results

Performance time was shorter in U compared to S (U: 17.73 ± 1.18 vs. S: 19.94 ± 1.41 s, *F*1,12 = 370.67, *p* < 0.01, η^2^ = 0.97, Table 1). Kicking frequency was increased in U compared to S (U: 135 ± 18 vs. S: 121 ± 15 kicks min^−1^, *F*1,12 = 13.47, *p* < 0.01, η^2^ = 0.53) and was increased in U compared to S during the last two sprints indicating interaction between conditions and repeated sprints (U: 122 ± 14, 133 ± 19, 144 ± 25, 141 ± 21; S: 122 ± 26, 124 ± 17, 122 ± 16, 116 ± 14 kicks∙min^−1^, *F*3,36 = 6.69, *p* < 0.05, η^2^ = 0.21, Table 1). The RPE increased with successive sprints reaching maximum values at the end of 4 x 50-m (U: 9 ± 1, S: 9 ± 1, *p* > 0.05), but the overall mean value was no different between conditions (U: 7 ± 2, S: 7 ± 2, *p* > 0.05, Table 1).

**Table 1:** Performance time, kicking frequency and rating of perceived exertion (RPE) values for the four 50-m sprints under both underwater and surface conditions. Values are mean ± SD.

UNDERWATER					
	1^st^ 50 m	2^nd^ 50 m	3^rd^ 50 m	4^th^ 50 m	MEAN
**Performance time (s)**	18.00 ± 1.17	17.22 ± 1.19	17.65 ± 1.12	18.03 ± 1.18	17.73 ± 1.18*
**Kicking frequency (kicks•min^−1^)**	122 ± 14	133 ± 19	144 ± 25#	141 ± 21#	135 ± 18*
**RPE** **(Borg 10)**	4 ± 1	6 ± 1#	8 ± 1#	9 ± 1#	7 ± 2
**SURFACE**					
	**1^st^ 50 m**	**2^nd^ 50 m**	**3^rd^ 50 m**	**4^th^ 50 m**	**MEAN**
**Performance time (s)**	20.04 ± 1.84	19.21 ± 1.27	20.08 ± 1.18	20.45 ± 1.08	19.94 ± 1.41
**Kicking frequency (kicks•min^−1^)**	122 ± 26	124 ± 17	122 ± 16	116 ± 14	121 ± 15
**RPE** **(Borg 10)**	4 ± 1	6 ± 2#	8 ± 1#	9 ± 1#	7 ± 2

* : significant difference between surface and underwater conditions, p < 0.05 #: significant difference between sprints, p < 0.05

**Figure 1:**
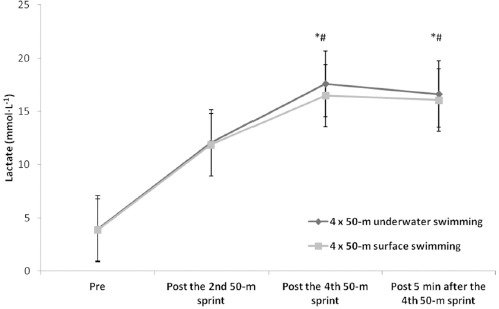
Lactate values for both surface and underwater conditions. Values are mean ± SD. *: significant difference compared to pre values under both conditions, p < 0.05. #: significant difference between the fourth sprint and five minutes during recovery compared to the second sprint under both conditions, p < 0.05

Mean and peak lactate concentrations were not different between conditions (peak lactate, U: 17.6 ± 3.5; S: 16.5 ± 3.1 mmol∙L^−1^, *p* > 0.05) and were increased following the fourth sprint and five minutes during recovery compared to the second sprint under both conditions (*F*1,12 = 101.58, *p* < 0.01, η^2^ = 0.89, Figure 1).

**Figure 2:**
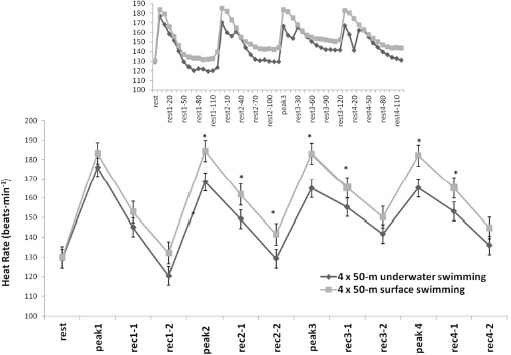
Heart rate values for both surface and underwater conditions. The inserted figure shows heart rate changes in 10-s intervals. Values are mean ± SD. *: significant differences between conditions, p < 0.01. Rest: the HR before the 1st sprint, Peak 1, Peak 2, Peak 3, Peak 4: indicate the highest HR at the end of the 1^st^, 2^nd^, 3^rd^, and 4^th^ sprints, respectively. Rec 1-1, Rec 1-2, and all other indices: indicate mean one-minute HR after each sprint in the first and the second minute into recovery. The first number indicates the sprint number and the second the minute or seconds (inserted figure) of recovery in successive sprints and minutes, respectively.

The mean heart rate during 4 x 50 m was decreased in U compared to S as it was also observed in the peak heart rate of each 50-m sprint (U: range 167–177 vs. S: range 183–185 b•min^−1^, *F*1,12 = 28.31, *p* < 0.01, η^2^ = 0.74, Figure 2). Interestingly, the heart rate during U showed a progressive decrease during the last 10 s of each sprint and remained at a lower level during 20 s of recovery compared to the S condition (U: range 141–151 vs. S: range 166–174 b•min^−1^, *F*12,120 = 105.71, *p* < 0.01, η^2^ = 0.91, Figure 2). The heart rate subsequently increased to a second peak within the first 30 s of recovery in U to reach values similar to the S condition.

Respiratory variables such as FEV1, FVC, PEF and MVV were not differed between the two conditions, however, FVC was decreased (*F*1,12 = 17.44, *p* < 0.01, η^2^ = 0.59) and MVV was increased (*F*1,12 = 6.54, *p* < 0.05, η^2^ = 0.35) post-sprints compared to starting values under both conditions (Table 2).

**Table 2:** Forced expiratory volume in 1 s (FEV1), forced vital capacity (FVC), peak expiratory flow (PEF) and maximal voluntary ventilation (MVV) values for the four 50-m sprints, pre and post under both underwater and surface conditions. Values are mean ± SD.

	UNDERWATER Pre	UNDERWATER Post	SURFACE Pre	SURFACE Post
**FEV1 (L)** **FVC (L)**	4.69 ± 0.59 5.51 ± 0.61*	4.70 ± 0.59 5.35 ± 0.70	4.76 ± 0.48 5.44 ± 0.55*	4.56 ± 0.73 5.26 ± 0.59
**PEF (L•s^−1^)**	8.83 ± 1.73	9.35 ± 1.91	9.40 ± 1.12	8.73 ± 1.70
**MVV (L•min^−1^)**	190.82 ± 23.97*	200.78 ± 25.99	191.48 ± 20.87*	199.28 ± 26.89

* Significant differences between pre and post under both conditions, p < 0.05

Finally, V̇O_2peak_, V̇CO_2_ production and V̇E were increased after the last sprint compared to the starting values (V̇O_2peak_: *F*1,12 = 454.91, *p* < 0.01, η^2^ = 0.98, V̇CO_2_: *F*1,12 = 226.95, *p* < 0.01, η^2^ = 0.96, V̇E: *F*1,12 = 292.13, *p* < 0.01, η^2^ = 0.97), but were not different between the two conditions (Table 3).

**Table 3:** Peak oxygen uptake (V̇O_2peak_), carbon dioxide production (V̇CO_2_) and pulmonary ventilation (V̇E) values for the four 50-m sprints, pre and post under both underwater and surface conditions. Values are mean ± SD.

	UNDERWATER Pre	UNDERWATER Post	SURFACE Pre	SURFACE Post
**V̇O_2peak_ (mL•kg^−1^•min^−1^)** **V̇CO_2_ (mL•min^−1^)**	6.0 ± 0.9* 382 ± 118*	67.7 ± 9.7 3326 ± 678	6.9 ± 1.9* 377 ± 1278*	65.3 ± 12.2 3663 ± 1089
**V̇E (L•min^−1^)**	18 ± 4*	141 ± 28	17 ± 4*	155 ± 41

* Significant differences between pre and post in both conditions, p < 0.05

## Discussion

The study analyzed the effects of maximal effort training on monofin swimmers, with and without apnea, on performance, lactate concentration, heart rate, perceived exertion, and respiratory variables. The main findings indicate improved underwater performance with increased kicking frequency despite similar metabolic responses between the two experimental conditions. Respiratory function was not different between conditions.

Performance time was shorter in U compared to S and 3–4 s slower compared to world records in the corresponding races, confirming the maximum effort and the high competitive level of participants. The U vs. S time differences are consistent with previous findings where performance time was significantly shorter in underwater compared to surface monofin swimming, possibly due to a lower active drag under the U condition (Nicolas and Bideau, 2009). This finding highlights the importance of optimizing swimming methods to exploit the advantages of underwater propulsion, thereby improving overall performance in competitive fin swimming events. Kicking frequency was the same in the first 50-m sprint, although it was increased in U compared to S during the last two sprints. A previous study indicated increased kicking frequency on the surface (Nicolas and Bideau, 2009). However, that study applied one single 25-m sprint monofin swimming compared to repeated 50-m sprints in the present study. The increased kicking frequency towards the last two 50-m sprints may be attributed to a combined effect of fatigue and personalized adjustments to maintain efficiency. This is supported by the observation that the preferred frequency is the most appropriate to maintain speed and any deviation will reduce the underwater speed (Vercruyssen et al., 2012). Monofin swimmers can enhance their performance by increasing their kicking frequency, as long as it can be adjusted to meet the metabolic energy demands in relation to exercise intensity and duration, regardless of their physical characteristics (Nicolas et al., 2007).

There were no significant differences in mean and peak lactate concentrations between the underwater and surface conditions (17.6 ± 3.5; 16.5 ± 3.1 mmol∙L^−1^, respectively). However, lactate concentration increased following the fourth sprint and during the five-minute recovery period compared to the second sprint under both conditions. The increasing kicking frequency appears to be a contributing factor for the increased lactate concentration at least under the U condition. However, the duration of each sprint was short and similar between conditions (18–20 s). Then, the duration difference between surface and underwater conditions may not be responsible for any differences in glycolytic contribution and this induced similar lactate response. Moreover, the 2-min recovery period between sprints did not allow for complete PCr resynthesis, thus increasing the reliance on glycolysis under both conditions (Bogdanis et al., 1998). In both cases, the metabolic needs were taxed to the maximum level despite differences in performance time.

Our findings are in line with the study of Guimard et al. (2014) who investigated the impact of a 100-m fin swimming exercise, divided into four 25-m segments, using either a breathing or breath-holding technique. That study found no significant difference in end-exercise blood lactate levels between the trials. However, it should be noted that the duration of swimming in that study was not enough to cause significant reliance on anaerobic glycolytic metabolism and the reported values were lower than in the present study (9 vs. 17 mmol∙L^−1^).

The mean heart rate was decreased in U compared to S as it was also observed in the peak heart rate of each 50-m sprint. No significant difference in the heart rate between 100-m fin swimming trials with and without breathing was been observed in a previous study (Guimard et al., 2014). However, in the present study, a lower heart rate was recorded in the underwater swimming condition compared to the surface swimming condition, despite the increased frequency of kicking and increased performance. This difference may be attributed to the swimming environment. Immersion in water activates the mammalian diving reflex, which triggers a series of physiological responses that help conserve oxygen and maintain vital functions during underwater immersion. These responses include bradycardia and peripheral vasoconstriction, among other adaptations that contribute to oxygen conservation. The lower heart rate observed during underwater fin swimming was due to bradycardia (Gooden, 1994). Increased bradycardia with face immersion during cycling at low intensity (80 W) compared to the same exercise intensity and free breathing has been reported previously (Wein et al., 2007).

The observed bradycardia increases along with the duration of apnea and is possibly greater during intense underwater exercise such as artistic swimming (Rodríguez-Zamora et al., 2012). The maximum intensity of exercise and apnea despite the short exercise duration applied in the present study probably induced severe bradycardia that recovered 20–30 s after the end of each sprint. The RPE values exhibited a consistent increase throughout successive sprints, culminating in peak values at the finish of the fourth 50-m bout (mean 9 ± 1). This observation is consistent with previous findings reported after the fourth 50-m swim in a set of 6 x 50 sprints using the same recovery period as applied in the present study (Kostoulas et al., 2018). Notably, the RPE values observed in the present study were recorded with a higher heart rate and lactate concentration compared to those previously reported (Kostoulas et al., 2018).

The study investigated the impact of intense fin swimming on respiratory function, focusing on FEV1, FVC, PEF, and MVV. Despite the consistency observed in FEV1, FVC, PEF and MVV across different experimental conditions, notable changes were detected in FVC and MVV values pre- and post-fin swimming bouts. These findings suggest a transient alteration in respiratory performance following intense fin swimming, which is indicative of the dynamic nature of the respiratory system's response to strenuous activities. Swimmers were of a high performance level in terms of their respiratory capacity, and their respiratory values in the present study were, on average, higher than in endurance athletes as reported in a previous study (Durmic et al., 2017), except for PEF (9.08 ± 1.62 vs. 9.20 ± 2.03 L•s^−1^). In contrast, higher values for swimmers' FEV1 (4.95 ± 0.42 L) and FVC (6.22 ± 0.60 L) compared to the present study were reported by Rosser-Stanford et al. (2019). FVC represents the maximum volume of air that can be forcefully expelled after a full inhalation. The reduction in FVC after vigorous exercise may result from a decrease in respiratory muscle strength following maximal incremental exercise, as observed in trained runners and cyclists (Oueslati et al., 2018). It is likely that increased blood flow due to the exercising leg muscles may degrease the diaphragmatic blood flow, thus contributing to respiratory muscles fatigue during heavy exercise (Vogiatzis et al., 2006). Therefore, we may expect that the increased leg work, as in the present study, would require more blood flow to the lower limbs, limiting its availability to respiratory muscles.

## Conclusions

The study compared the effects of two training conditions, U and S, on performance, physiological responses, and rating of perceived exertion variables during repeated 50-m sprints. The results showed that performance time was better in U monofin swimming compared to S, with a notable progressive increase in kicking frequency at the end of repeated efforts. Although the peak lactate concentration and the RPE did not differ, the heart rate was lower in U monofin swimming. The pulmonary variables were comparable between the training protocols, with a significant change observed from pre- to post- efforts, including a decrease in FVC and an increase in MVV values. These findings indicate that underwater monofin training may result in enhanced performance and maximized physiological responses compared to surface training during repeated sprint exercises.
